# TRPV4 activation in Schwann cells mediates mechanically induced pain of oral cancer

**DOI:** 10.3389/fpain.2025.1532885

**Published:** 2025-03-12

**Authors:** Yatendra Mulpuri, Nguyen H. Tu, Kenji Inoue, Grace Harden, Samuel J. Nicholson, Anisa Seenauth, Yan Huang, Keylin G. Escobar, Yalda Moayedi, Nigel W. Bunnett, Donna G. Albertson, Brian L. Schmidt

**Affiliations:** ^1^ NYU Dentistry Translational Research Center, New York University Dentistry, New York, NY, United States; ^2^ NYU Pain Research Center, New York University, New York, NY, United States; ^3^ Department of Molecular Pathobiology, New York University Dentistry, New York, NY, United States; ^4^ Neuroscience Institute, New York University Langone Health, New York, NY, United States; ^5^ Department of Neuroscience and Physiology, New York University Grossman School of Medicine, New York, NY, United States

**Keywords:** oral cancer, pain, TRPV4, Schwann cells, mechanical hypersensitivity

## Abstract

**Introduction:**

Patients with oral cancer often experience intense functional pain due to mechanical stimulation at the cancer site. The role of mechanosensitive ion channels in oral cancer pain, such as TRPV4, is not fully understood.

**Objectives:**

Our objective was to investigate the role of Schwann cell TRPV4 in oral cancer pain.

**Methods:**

We examined the impact of TRPV4 inhibition on oral cancer pain in NU/J and C57BL/6J mice injected with human tongue cancer cell line (HSC-3) and mouse oral cancer cell line (MOC2) in the hind paw or tongue. Mechanical and heat sensitivity were assessed using the von Frey and Hargreaves tests, respectively. TRPV4 expression and functional activity in Schwann cells were analyzed using immunohistochemistry, qRT-PCR, Ca^2+^ imaging, and patch-clamp electrophysiology. The effect of TRPV4 activation on Schwann cell responses to mechanical stimulation was evaluated using a piezo stimulator. Conditioned media (CM) from TRPV4-activated Schwann cells were injected into the mouse paw to evaluate the contribution of TRPV4 in Schwann cells to mechanical hypersensitivity.

**Results:**

TRPV4 inhibition reduced paw cancer mechanical nociception in mice dose-dependently without affecting heat sensitivity. TRPV4 inhibition also decreased facial nociception in tongue cancer mice. TRPV4 was expressed mainly on the plasma membrane of mouse Schwann cells and activation of TRPV4 induced Ca^2+^ responses and whole-cell membrane currents in human Schwann cells. Mechanoactivated currents in human Schwann cells were inhibited by the TRPV4 antagonist HC-067047. Schwann cell CM induced mechanical hypersensitivity in mice, which was blocked by pre-treatment with HC-067047.

**Conclusion:**

TRPV4 activation plays a role in mediating mechanically induced pain of oral cancer.

## 1 Introduction

Oral cancer patients often endure intense pain caused by mechanical stimulation at the cancer site (1, 2). Pain interferes with daily functions, such as talking and eating, particularly when the cancer site comes into contact with food or teeth. Orofacial movements that involve pressure and stretching of the cancer at soft tissues, such as the tongue and buccal mucosa, can also trigger severe pain. Opioid analgesics have limited effectiveness because increasing doses are required as tolerance develops, leading to undesirable side effects.

Mechanosensitive ion channels, including transient receptor potential cation channel subfamily V member 4 (TRPV4), piezo type mechanosensitive ion channel component 1 (Er blood group) (PIEZO1), and piezo type mechanosensitive ion channel component 2 (PIEZO2), are activated by pressure, stretch, and tactile stimuli (3–5). TRPV4, PIEZO1, and PIEZO2 channels have been implicated in pathological pain (6–8). The expression of PIEZO1 and PIEZO2 in neurons is relatively high compared to TRPV4, particularly in neurons that mediate low-threshold mechanosensation (4, 9–11). TRPV4, PIEZO1, and PIEZO2 channels are expressed in various cell types, including epithelial cells (12–14), fibroblasts (15–17), neurons (9, 11, 18), and Schwann cells, the glial cells of the peripheral nervous system (19, 20).

Cancer cells and Schwann cells reciprocally interact to promote cancer growth (21, 22). Oral cancer-activated Schwann cells induce nociceptive behaviors in mice, which has been attributed to the release of nociceptive mediators from the activated Schwann cells (22, 23). The role of TRPV4 in Schwann cells in relation to pain has not been explored. Here, we investigated whether TRPV4 is involved in oral cancer pain. We found that antagonism of TRPV4 reduced mechanical hypersensitivity in oral cancer pain models and that conditioned media (CM) from TRPV4-activated Schwann cells induced hypersensitivity to mechanical stimulation. Inhibition of TRPV4 attenuated Schwann cell responses to mechanical stimulation.

## 2 Materials and methods

Information on the key resources is provided in Supplementary Table S1.

### 2.1 Animals

Male C57BL/6J and NU/J mice, 4–8 weeks old, were used in experiments. All mice were housed five per cage in the vivarium under a 12 h light/dark cycle and had *ad libitum* access to food and water. After each experiment, all mice were humanely euthanized using 100% carbon dioxide at a fill rate of 60% chamber volume displacement per minute for 6 min, followed by cervical dislocation. The New York University (NYU) Institutional Animal Care and Use Committee approved the mouse studies (protocol # IA16-00437, date of approval 23 May 2023).

### 2.2 Cells and cell lines

The human tongue cancer cell line (HSC-3) human oral tongue cancer cell line (RRID: CVCL_1288) was obtained from the Japanese Collection of Research Bioresources Cell Bank (Catalog #JCRB0623, FUJIFILM Irvine Scientific, Santa Ana, CA, USA). The cells were maintained in Eagle's Minimum Essential Medium with 10% FBS and used in experiments at Passage 5. The mouse oral cancer cell line (MOC2) murine oral cavity cancer cell line (RRID: CVCL_ZD33) was purchased from Kerafast (Catalog #EWL002-FP, Kerafast, Boston, MA, USA) and was maintained in HyClone Iscove's Modified Dulbecco's Medium supplemented with Ham's nutrient mixture F12, 5% FBS, insulin (10 mg/ml), hydrocortisone, and EGF. MOC2 cells were used for inoculation at Passage 5.

Primary mouse Schwann cells were purchased from two sources and included Schwann cells isolated from the sciatic nerve of postnatal day 8 C57BL/6 mice (Catalog #M1700-57, ScienCell Research Laboratories, Carlsbad, CA, USA), spontaneously immortalized mouse Schwann cells originally isolated from dorsal root ganglia and spinal nerves of C57BL/6 mice (Catalog #1970C3, Applied Biological Materials, Inc., Richmond, BC, Canada). Primary human Schwann cells isolated from human spinal nerve were purchased from Neuromics, Inc. (Catalog #HMP303, Neuromics, Inc., Minneapolis, MN, USA). The human Schwann cells were cryopreserved at Passage 1 and used in experiments between Passage 4 and Passage 9. The commercial supplier of the primary human Schwann cells attests that the tissue was obtained under informed consent and with full compliance to appropriate provisions in accordance with applicable laws and regulations regarding the ethical collection of human specimens for biomedical research. Cells were maintained in Schwann cell medium for the duration of the experiments.

### 2.3 Drugs and chemicals

GSK2193874 was dissolved in 5% Tween 80% and 2% DMSO in DPBS. Stock solutions of GSK1016790A and HC-067047 were prepared in DMSO. All chemicals were purchased from Sigma-Aldrich (St. Louis, MO, USA) unless otherwise specified.

### 2.4 Mouse oral cancer models

At the beginning of each experiment, mice were randomly assigned to the experimental groups by an investigator blinded to the experiments using a sequence of random numbers. Paw cancer xenograft and tongue cancer allograft models were generated as described (24, 25). Mice were sedated with 4% isoflurane in 1 L/min of medical-grade oxygen prior to cancer cell line inoculation. The HSC-3 paw cancer xenograft model was generated in NU/J mice. The plantar surface of the left hind paw was disinfected with a PDI Alcohol Prep Pad and inoculated with 2 × 10^5^ HSC-3 cells in 20 μl of DMEM and Matrigel (1:1 in volume) into the mid-plantar area using a Lo-Dose U-100 Insulin Syringe. The tongue cancer model was generated in C57BL/6J mice by inoculating 5 × 10^3^ MOC2 cells in DMEM and Matrigel (1:1 in volume) into the middle of the tongue. After the injections, the mice were returned to their home cages and monitored for the first 3 days post-inoculation and then every week after injection for any injection-related complications.

### 2.5 Hind paw mechanical nociception

The von Frey test was used to measure hind paw mechanical nociception in NU/J mice with paw cancer (24). Mice were placed in individual, transparent enclosures (L × W × H, 6 × 2 × 2 inches) standing upon an elevated wire-mesh floor. The mice were acclimated for 1 h every 3 days for 2 weeks and before each assay. Calibrated von Frey filaments were inserted through the mesh floor to apply mechanical stimulation to the middle plantar surface of the left hind paw. The up-down method of Chaplan et al. (26) was used to determine the paw withdrawal threshold. The withdrawal threshold for each mouse was measured as the mean of three trials taken at least 5 min apart.

### 2.6 Hind paw thermal nociception

The Hargreaves test was performed using a paw thermal stimulator to measure thermal hyperalgesia in NU/J mice with paw cancer as described previously (24). Thermal withdrawal latencies were measured by focusing a radiant heat light with a spot area of 4 × 6 mm on the plantar surface of the hind paw. The heat stimulator was set to 35.0°C for the first stimulation and increased at the rate of 2.5°C/s with a cutoff latency of 10 s. Three trials were performed at least 5 min apart. Paw withdrawal latency was measured as the mean of the three trials.

### 2.7 Facial mechanical nociception

Facial mechanical sensitivity in mice with tongue cancer was measured with von Frey filaments as described (24, 27). The von Frey filament was applied to the cheek, and a response score was measured for each application. The facial nociception score was reported as a numerical average of the 11 responses in the following response categories: 0, no response; 1, detection (the mouse is aware of the filament that stimulates the face; the mouse turns its head slightly to the object); 2, reaction (the mouse turns its head away quickly, pulls it backward, or reacts with a single face wipe); 3, escape/attack (the mouse quickly escapes from the object, attacks the object with its paw or mouth, or reacts with two facial wipes); 4, multiple facial grooming (the mouse responds to the filament simulation with more than three facial wipes continuously) (24).

The investigators involved in the behavioral testing of the animals were blinded to the drug treatments.

### 2.8 Quantitative reverse transcription PCR (qRT-PCR)

Primary mouse Schwann cells were plated at 5 × 10^5^ cells/well in a 6-well plate coated with type I collagen and cultured for 10 days until cells became 80%–90% confluent. Total RNA was isolated using an RNeasy Mini Kit following the manufacturer's instructions. RNA concentrations were measured using a NanoDrop Spectrophotometer. Complementary DNA (cDNA) was synthesized using a High-Capacity cDNA Reverse Transcription Kit. The first-strand cDNA synthesis mix consisted of 1 μg of total RNA, random hexamer primers, and RT reaction master mix for a total volume of 20 μl as recommended by the manufacturer. SYBR green-based qRT-PCR mixture contained a cDNA template, primers, and PowerUp SYBR Green Master Mix. The reaction was run on an AriaMx real-time PCR system. The cycling conditions included an initial step at 50°C for 2 min, pre-denaturation at 95°C for 2 min, and 40 cycles at 95°C for 15 s and at 60°C for 1 min. The threshold detection cycle of each gene was determined using AriaMx real-time PCR software. Gene expression was normalized to the internal control *Gusb* using the 2^−ΔΔCt^ method. Primer sequences were obtained from PrimerBank (28, 29) and synthesized by IDT. The primers used to evaluate gene expression are shown in Supplementary Table S2.

### 2.9 Immunohistochemical (IHC) staining of TRPV4 in Schwann cells

Spontaneously immortalized mouse Schwann cells were cultured on type I collagen-coated culture plates for 10 days until cells reached 90% confluency. Adherent Schwann cells were released by incubating with trypsin-EDTA (0.025%, 37°C, 5 min) and rinsed twice in DPBS. Cells were pelleted by centrifugation (1,000 rpm, 5 min) and incubated in 10% neutral buffered formalin at 4°C overnight. The cell pellet was embedded in agarose and paraffin and sectioned. Samples were immunostained on a Leica BOND RX automated stainer, according to the manufacturer's instructions. In brief, tissues underwent deparaffinization online, followed by epitope retrieval for 60 min at 100°C with Leica Biosystems ER2 solution (pH 9) and endogenous peroxidase activity blocking with H_2_O_2_. Sections were then incubated with primary antibody against TRPV4 (Catalog #ab231772, Abcam, Waltham, MA, USA) at 1:100 for 60 min at room temperature. Primary antibodies were detected with anti-rabbit HRP-conjugated polymer and 3,3′-diaminobenzidine substrate that is provided in the Leica BOND Polymer Refine Detection System, followed by counterstaining with hematoxylin. Slides were scanned at 40× on a Hamamatsu NanoZoomer 2.0 HT, and the image files were uploaded to the NYU Grossman School of Medicine OMERO Plus image data management system. The percent of Schwann cells that express TRPV4 was analyzed using National Institutes of Health ImageJ software. Cells were analyzed from five randomly selected regions of interest (ROI). A multi-point selection tool was used to measure the total number of Schwann cells per ROI (range, 266–343 cells/ROI) and the percent of Schwann cells that express TRPV4 above the set threshold intensity.

### 2.10 Microplate-based ratiometric calcium imaging

Human Schwann cells were seeded in type I collagen-coated 96-well plates and incubated until they had reached 80%–90% confluency. For ratiometric measurement of intracellular Ca^2+^, cells were washed in assay buffer (150 mM NaCl, 2.6 mM KCl, 2.2 mM CaCl_2_, 1.18 mM MgCl_2_, 10 mM D-glucose, 10 mM HEPES, pH 7.4) containing 4 mM probenecid and 0.5% BSA and then incubated with Fura-2 AM (1 μM) in assay buffer for 30 min at 37°C. The Fura-2 AM was aspirated, and cells were washed with assay buffer twice and allowed to incubate for 10 min at 37°C. Fura 2-AM fluorescence was measured with a FlexStation 3 (Molecular Devices) using 340/380 nm excitation and 530 nm emission. Fluorescence was measured at 5 s intervals. The results were baseline corrected and expressed as a change in 340/380 nm.

### 2.11 Electrophysiology

For whole-cell patch-clamp recordings, human Schwann cells were plated on 5 mm round glass coverslips coated with poly-D-lysine and type I collagen and maintained in a humidified atmosphere at 37°C for 24 h. All patch-clamp recordings were performed at room temperature (∼24°C). The bath solution contained 143 mM NaCl, 5 mM KCl, 2 mM CaCl_2_, 2 mM MgCl_2_, 10 mM glucose, and 10 mM HEPES (pH 7.4; 310 mOsm). The pipette solution contained 142 mM CsCl, 1 mM CaCl_2_, 1 mM MgCl_2_, 5 mM EGTA, 10 mM HEPES, 2 mM Mg-ATP, and 0.2 mM Na-GTP (pH 7.2; 290 mOsm). Patch electrodes were pulled from borosilicate glass on a micro-pipette puller and had a resistance of 5–7 MΩ. The recording chamber was continuously perfused with the external solution at 1 ml/min. Pipette capacitance was neutralized after a giga-ohm seal was made, and whole-cell capacitance was compensated after achieving the whole-cell configuration. Whole-cell membrane currents were recorded in voltage-clamp mode after a 3 min equilibration. Schwann cell membrane currents were recorded every 20 s using a voltage-ramp protocol (−100 to +100 mV, 500 ms) from a holding potential of 0 mV. Currents were filtered at 2 kHz and digitized at 10 kHz. All recordings were performed using an Axopatch 200B amplifier and Digidata 1440A digitizer, and data were acquired and analyzed using pCLAMP 10 software.

Mechanoactivated currents were recorded from human Schwann cells voltage clamped at −60 mV. The Schwann cell membrane was mechanically stimulated with a blunt glass probe (tip diameter, 2–3 µm) mounted on a micromanipulator at a 45° angle and positioned close to the plasma membrane without touching the membrane. Using a piezo servo controller, the stimulating probe was advanced in 1 μm increments (0–5 μm) with a square-pulse protocol at 15 s intervals, each step lasting 300 ms. The mechanical stimulation was automated with Digidata 1440A, and signals were amplified on Axopatch 200B. Cells with access resistance >10 MΩ and that did not maintain a giga-ohm seal during mechanical stimulation were excluded from the analysis.

### 2.12 Schwann cell-conditioned medium (CM) collection and nociception testing

Primary mouse Schwann cells (1 × 10^5^ cells/ml) were cultured in type I collagen-coated 6-well culture plates for 10 days until cells reached 80%–90% confluency. Schwann cell medium was removed. Cell cultures were rinsed twice with DPBS and incubated with an external medium (143 mM NaCl, 5 mM KCl, 2 mM CaCl_2_, 2 mM MgCl_2_, 10 mM glucose, and 10 mM HEPES; 0.2 µm sterile filtered) containing vehicle DMSO (0.001%) or HC-067047 (3 µM and 10 µM) for 15 min followed by incubation with vehicle or GSK1016790A (0.3 µM) in external medium for 105 min. The Schwann cell CM was collected and transferred to high-flux polyethersulfone (PES) membrane protein concentrators with a molecular weight cutoff of 3 kDa to filter out the drugs. The PES concentrators were centrifuged at 3,000 × *g* for 60 min at 24°C until the sample volume was reduced by 95%. The Schwann cell CM was resuspended every 20 min to prevent clogging of the PES membrane pores. Filtered CM was injected in the hind paw of mice immediately after collection for mechanical nociception testing. The investigator conducting behavioral testing on the animals was blinded to the drugs and CM information.

### 2.13 Statistical analysis

GraphPad Prism was used for the statistical analysis. The results were expressed as mean ± standard error of the mean (SEM). Differences were evaluated by unpaired *t*-test or one- or two-way ANOVA with Šídák's or Tukey's multiple-comparisons tests.

## 3 Results

### 3.1 TRPV4 inhibition in oral cancer alleviates mechanical nociception, but not thermal nociception

**Figure 1:**
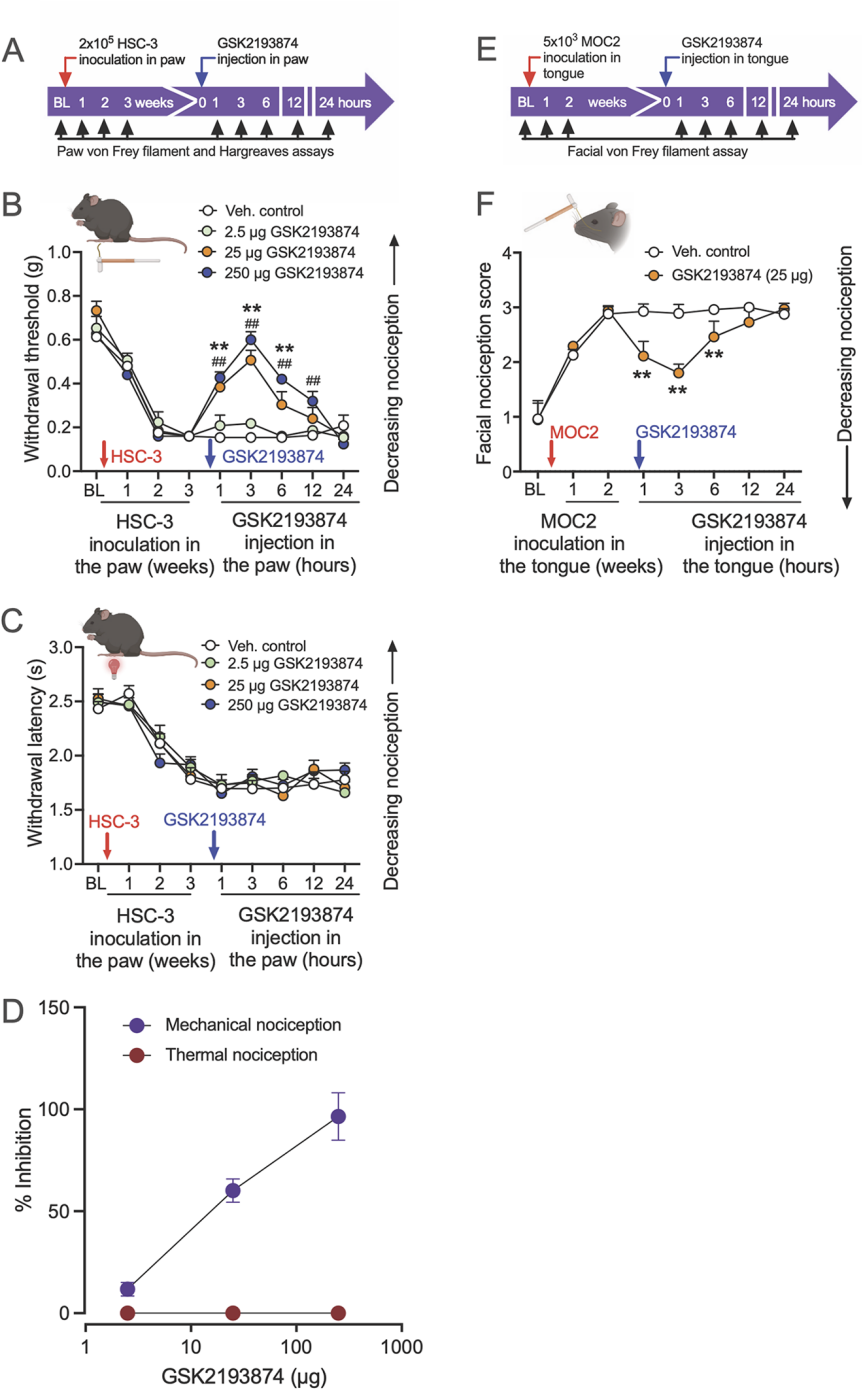
Pharmacological inhibition of TRPV4 attenuates oral cancer-induced mechanical nociception, but not thermal nociception, in paw and tongue oral cancer mouse models. **(A)** The experimental timeline indicates baseline measurements of mechanical and thermal nociception in the hind paw of NU/J mice, HSC-3 inoculation, TRPV4 antagonist administration, and nociception testing. **(B)** HSC-3 inoculation in the hind paw (red arrow) produced mechanical allodynia after 2 weeks. GSK2193874 administration (blue arrow) dose-dependently reversed mechanical allodynia measured at 1, 3, 6, 12, and 24 h after the injections [dose *F*_(3, 144)_ = 31, *p* < 0.001; ^##^*p* < 0.01 at 1, 3, 6, and 12 h when mice treated with 250 µg GSK2193874 are compared with vehicle-treated mice (Veh., control); **p < 0.01 at 1, 3, and 6 h when mice treated with 25 µg GSK2193874 are compared with vehicle-treated mice, *n* = 5 mice/group, two-way ANOVA, Tukey's multiple-comparisons test]. **(C)** HSC-3 inoculation produced thermal hyperalgesia after 3 weeks. GSK2193874 administration in the paw had no effect on thermal withdrawal latencies measured at all time points tested. **(D)** Dose–response of percent inhibition by GSK2193874 on peak mechanical and thermal nociception (3 h post-injection) in HSC-3 inoculated mice. Percent nociception inhibition was calculated using the formula: {(post-drug response at 3 h – pre-drug response at Week 3)/[pre-cancer response (baseline) – pre-drug response at Week 3]} × 100. A response is defined as a mechanical withdrawal threshold or thermal withdrawal latency. **(E)** The experimental timeline indicates baseline measurements of facial nociception, MOC2 inoculation in the tongue of C57BL/6J mice, GSK2193874 administration in the tongue, and facial nociception testing. **(F)** MOC2 inoculation increased facial nociception score higher than baseline at 1 and 2 weeks; GSK2193874 (25 µg) administration reversed facial nociception at 1, 3, and 6 h after the injection (treatment *F*_(1, 62)_ = 48.4, *p* < 0.0001; **p < 0.01 vs. vehicle control, *n* = 5 mice/group, two-way ANOVA, Tukey's multiple-comparisons test). Data are presented as mean ± SEM. Images in **B**, **C**, and **F** were created with BioRender.com.

To examine the involvement of TRPV4 in oral cancer-related pain, we investigated whether antagonism of TRPV4 would reduce mechanical allodynia and thermal hyperalgesia evoked by the growth of oral cancer cells in the mouse hind paw. After establishing baseline mechanical withdrawal thresholds and thermal latencies in the hind paw of NU/J mice, we inoculated HSC-3 cells in the left hind paw, which led to increased mechanical and thermal nociception over 3 weeks (Figures 1A–D). Subsequently, we administered vehicle or different doses of a highly selective TRPV4 antagonist, GSK2193874 (2.5, 25, and 250 µg), into the cancer model, and measured the effect on oral cancer-induced mechanical allodynia and thermal hypersensitivity. GSK2193874 at doses of 25 and 250 µg reduced oral cancer-induced mechanical allodynia at 1, 3, 6, and 12 h post-injection (Figures 1B,D). In contrast, GSK2193874 did not show an effect on thermal withdrawal latencies at the measured time points (Figures 1C,D).

We also examined the effect of TRPV4 inhibition on orofacial mechanical nociception measured with von Frey filaments in the tongue cancer model. After baseline facial nociception scores were measured, C57BL/6J mice were inoculated with MOC2 oral cancer cells in the tongue. Upon reaching peak nociception scores at 2 weeks post-inoculation, mice were injected with vehicle or GSK2193874 (25 µg) in the tongue, and facial nociception was measured at 1, 3, 6, 12, and 24 h. Facial nociception was reversed in mice injected with GSK2193874 at 1, 3, and 6 h (Figures 1E,F). These results suggest that TRPV4 activation mediates mechanically induced oral cancer nociception.

### 3.2 TRPV4 is expressed on the plasma membrane of Schwann cells

**Figure 2:**
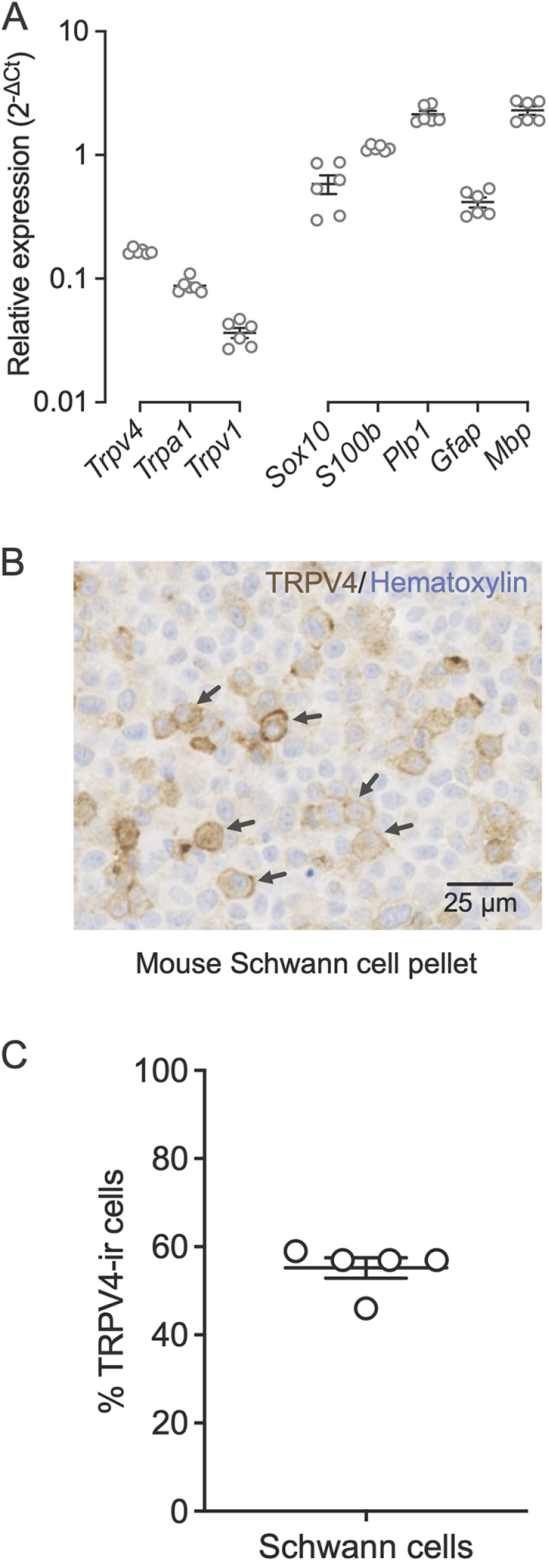
Schwann cells express TRPV4 on the plasma membrane. **(A)** Relative gene expression levels of *Trpv4*, *Trpa1*, *Trpv1*, and Schwann cell markers, *Sox10*, *S100b*, *Plp1*, *Gfap*, and *Mbp* in mouse primary Schwann cells measured with qRT-PCR. Gene expression was normalized to the internal control *Gusb* using the 2^−ΔΔCt^ method. Data are presented as mean ± SEM. **(B)** Immunohistochemical staining of TRPV4 in formalin-fixed and paraffin-embedded sections of a mouse Schwann cell pellet. The arrows indicate the TRPV4 immunoreactivity on the Schwann cell membrane. **(C)** Percent of Schwann cells that are immunoreactive for TRPV4 measured in five randomly selected fields from the total scan area. The average number of Schwann cells measured per field is 315.4 ± 16.8. Data are presented as mean ± SEM.

We hypothesized that TRPV4, a mechanosensitive ion channel (5, 30), mediates oral cancer mechanical nociception. Therefore, we confirmed that TRPV4 is expressed in Schwann cells. We extracted RNA from primary cultures of mouse Schwann cells isolated from sciatic nerves and measured gene expression by qRT-PCR (Figure 2A). We established that the cultured cells expressed the Schwann cell markers (*Sox10*, *S100b*, *Plp1*, *Gfap*, and *Mbp*). *Trpv4* was more highly expressed than transient receptor potential cation channel subfamily A member 1 (*Trpa1*) and transient receptor potential cation channel subfamily V member 1 (*Trpv1*), channels associated with oral cancer pain in neurons (31, 32). TRPV4 immunoreactivity was observed on the plasma membrane in 55.2% ± 2.3% of the Schwann cells (Figures 2B,C).

### 3.3 TRPV4 is functionally expressed on human Schwann cells

**Figure 3:**
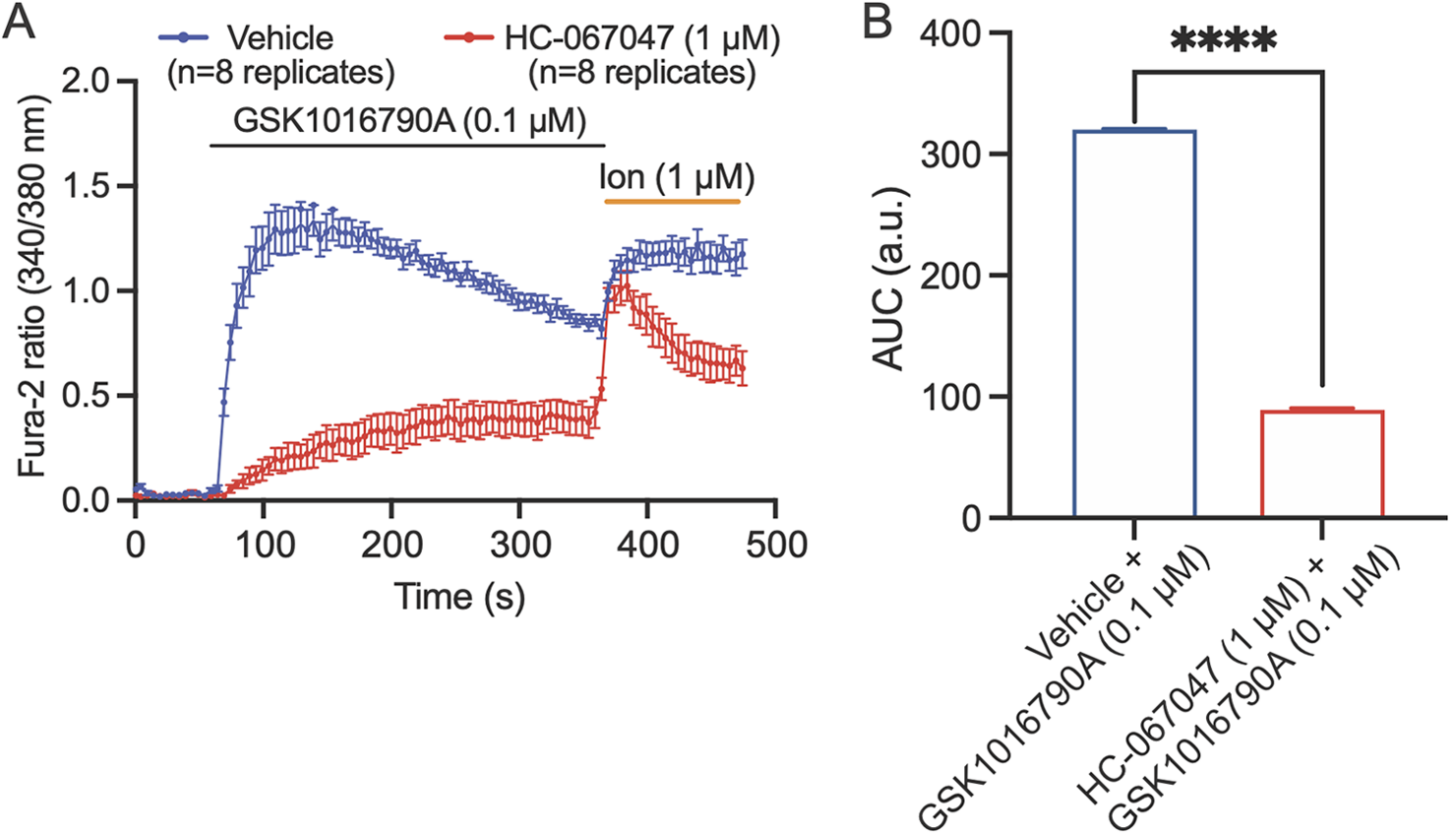
TRPV4 is functionally expressed by human Schwann cells. **(A)** Kinetic plots showing the responses of Schwann cells to stimulation with TRPV4 agonist GSK1016790A. Schwann cells were pre-treated with vehicle (blue) or TRPV4 antagonist, HC-067047 (1 µM, red) for 30 min, and baseline recordings were collected for 60 s before addition of the TRPV4-selective agonist GSK1016790A (0.1 µM). After 360 s, 1 µM ionomycin (ion) was applied to confirm cell viability. **(B)** The area under the curve (AUC) of the kinetic plots in **A**. ****p < 0.0001, unpaired *t*-test. Data are presented as mean ± SEM, *n* = 8 technical replicates/group.

We investigated whether TRPV4 is functionally expressed on cultured human Schwann cells by measuring calcium flux in response to TRPV4 agonists and antagonists using a microplate-based ratiometric calcium assay with Fura-2. Cells were pre-treated with vehicle (control) or TRPV4 antagonist, HC-067047 (1 µM) for 30 min prior to imaging. Application of GSK1016790A (0.1 µM) to human Schwann cells, cultured to 90% confluency, elicited a robust intracellular Ca^2+^ response that peaked 5 s after the initial stimulation and was maintained until the addition of ionomycin (1 µM). The response was significantly reduced when cells were pre-incubated with the TRPV4 antagonist HC-067047 (1 µM) for 30 min prior to application of GSK1016790A (0.1 µM) (Figures 3A,B), demonstrating that the observed Schwann cell responses were TRPV4 dependent.

**Figure 4:**
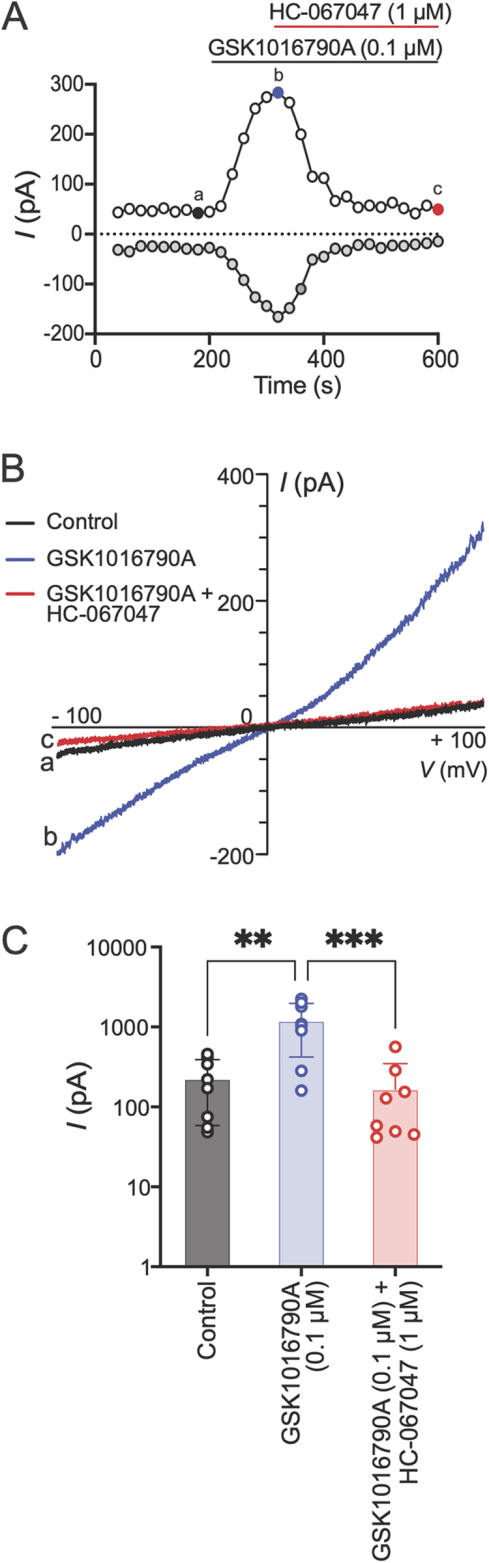
TRPV4 activation mediates Schwann cell membrane currents. **(A)** Time course of human Schwann cell membrane currents measured at baseline, after application of GSK1016790A (0.1 µM) and co-application of GSK1016790A with HC-067047 (1 µM). The ramp pulses were applied every 20 s from −100 to +100 mV for 500 ms from a holding potential of 0 mV. The white and gray dots represent current amplitudes measured at +90 and −90 mV membrane potentials, respectively. **(B)** Representative current–voltage curves taken at time points a, b, and c (color coded) in **A**. **(C)** Peak current amplitudes measured at +90 mV under baseline condition (control), at peak response after GSK1016790A application and co-application of GSK1016790A with HC-067047. Each dot represents peak current amplitude measured from one Schwann cell (**p < 0.01, ***p < 0.001; *n* = 8 cells from two independent experiments, one-way ANOVA, Tukey's multiple-comparisons test). Data are presented as mean ± SEM.

To further examine if the Schwann cell responses in Ca^2+^ imaging were due to TRPV4 activity on the plasma membrane, we measured Schwann cell membrane currents in response to TRPV4 agonist application using whole-cell patch-clamp electrophysiology. GSK1016790A (0.1 µM) was applied to the bath for 2 min followed by co-application of GSK1016790A with an antagonist (HC-067047, 1 µM) for 5 min. Application of GSK1016790A (0.1 µM) induced an outwardly rectifying TRPV4-like current (33). These responses were abolished when GSK1016790A was co-applied with the TRPV4 antagonist HC-067047 (1 µM) (Figures 4A–C). These results demonstrate that human Schwann cells express functional TRPV4 on the plasma membrane.

### 3.4 TRPV4 inhibition reduces mechanoactivated currents in Schwann cells

**Figure 5:**
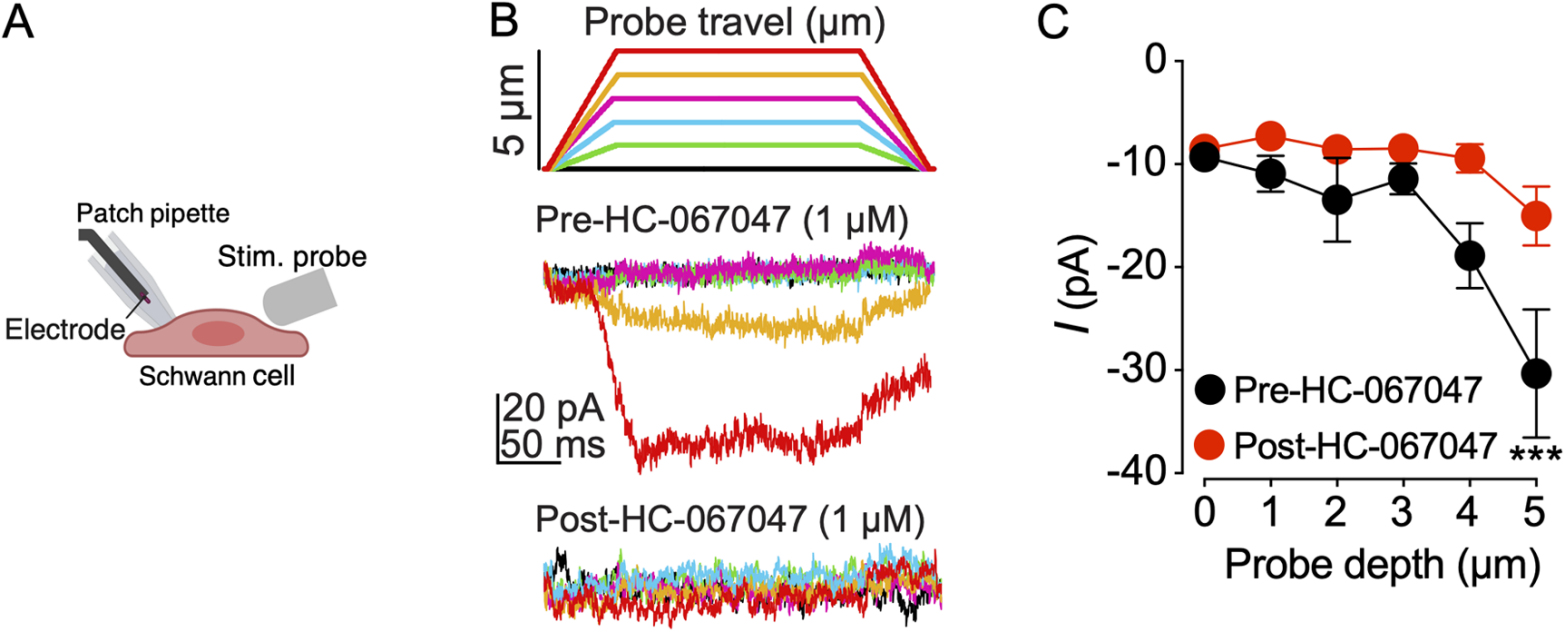
TRPV4 inhibition decreases mechanoactivated currents in human Schwann cells. **(A)** Schematic of mechanical stimulation of a human Schwann cell with a blunt glass probe (Stim. probe). Image created in BioRender.com. **(B)** Schematic of indentation steps illustrating probe travel with each depth indicated by a color. Representative whole-cell patch-clamp recordings of Schwann cell membrane currents elicited by mechanical stimulation at −60 mV. Schwann cells were stimulated by advancing the probe in 1 µm increments every 15 s before and after a 5 min bath application of TRPV4 antagonist, HC-067047 (1 µM). Current traces are color-matched to the probe depth of the stimulating glass probe. **(C)** Summary of the effect of HC-067047 on Schwann cell mechanoactivated currents. ***p = 0.0006 at 5 µm probe travel when current amplitudes are compared for pre-HC-067047 vs. post-HC-067047 addition, two-way ANOVA (*n* = 9 cells), Šídák's multiple-comparisons test. Data are presented as mean ± SEM.

Mechanically induced pain is a significant concern among oral cancer patients. To investigate whether TRPV4 activation on Schwann cells mediates mechanosensitivity, human Schwann cells were mechanically stimulated using a blunt glass probe driven by a piezo stimulator, and whole-cell membrane currents were recorded with patch-clamp technique. Mechanoactivated currents were recorded before and after a 5 min bath application of the TRPV4 antagonist (HC-067047, 1 μM). The results showed that mechanical stimulation with a glass probe induced currents in Schwann cells, and the amplitude of these mechanoactivated currents increased with the depth of the probe stimulation. These Schwann cell currents were attenuated by subsequent application of the TRPV4 antagonist HC-067047 applied for 5 min (Figures 5A–C), demonstrating that the Schwann cell response was mediated by TRPV4.

### 3.5 Contribution of TRPV4 on other cell types to oral cancer pain

We have demonstrated that *Trpv4* is expressed on mouse Schwann cells and *TRPV4* is functionally expressed on human Schwann cells. *TRPV4* is expressed in many cell types, including epithelial cells (12), fibroblasts (15), and neurons, in addition to Schwann cells. Current thinking about oral cancer pain has focused on the sensitization of neurons by pain mediators released in the cancer microenvironment. Immunohistochemical analysis of TRPV4 expression in mice bearing tongue cancer xenografts or allografts revealed TRPV4 immunoreactivity (ir) on the cell membranes of the tongue epithelium, ducts of the von Ebner glands and blood vessels (Supplementary Figure S1), as well as mouse cancer cells. TRPV4-ir was not detected in the nerve trunks carrying myelinated nerves. Membranous TRPV4-ir was also not detected in trigeminal ganglia (TG) from the tongue cancer mice. To mirror the detection of TRPV4 in cultured Schwann cells, we prepared cell pellets from TG neuron cultures, as we did for the cultured Schwann cells. TRPV4-ir was not detected on the membrane of the cells in the TG neuron pellet. In addition, only low levels of *Trpv4* mRNA in mouse TG sections were identified in 4.0% ± 0.6% of neurons by RNAscope (Supplementary Figure S2). Thus, TRPV4 on Schwann cells is likely to play a greater role in mediating oral cancer pain than TRPV4 on neurons.

### 3.6 Schwann cell mediators released by TRPV4 activation evoke mechanical nociception

**Figure 6:**
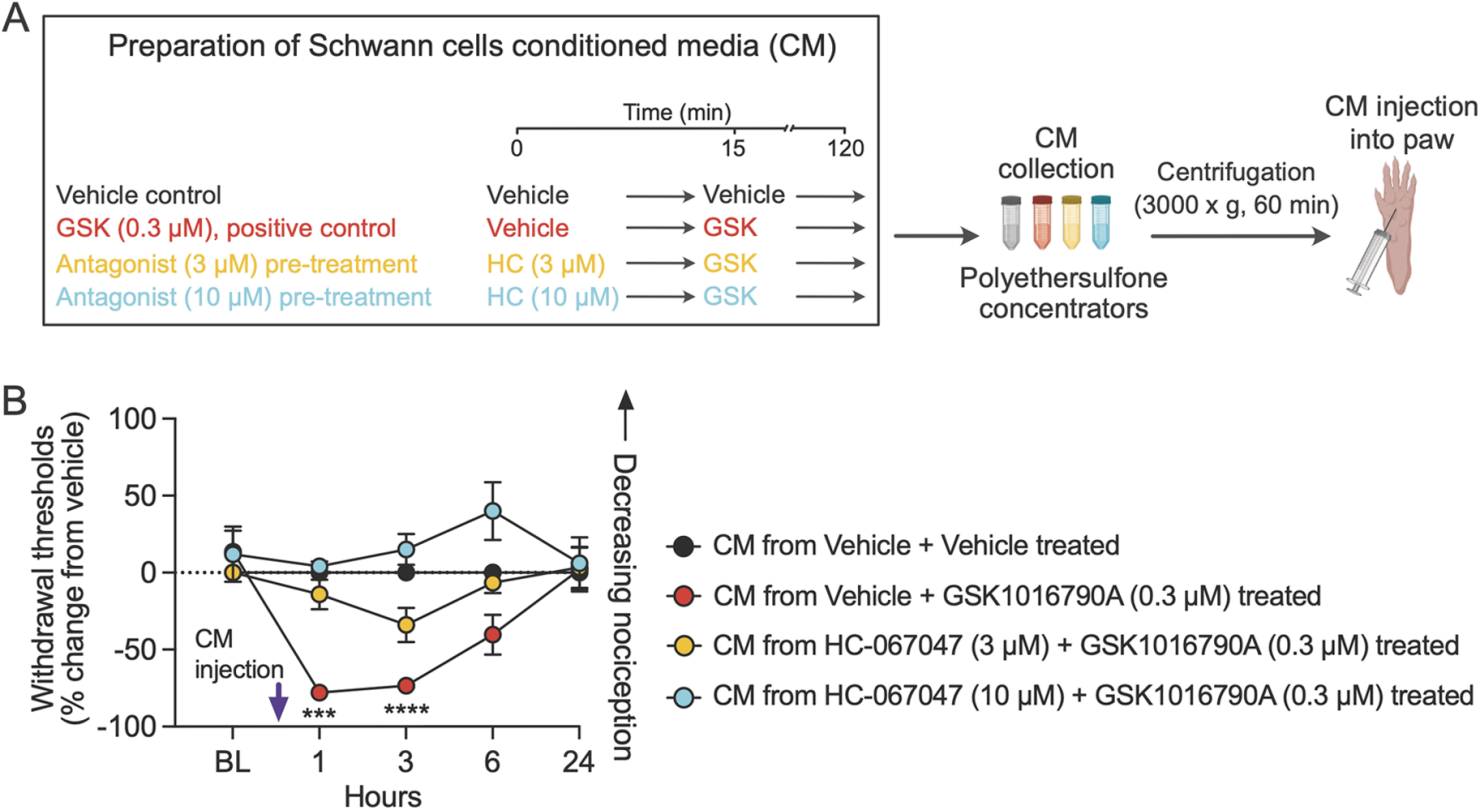
TRPV4 activation in Schwann cells induces the release of mediators that evoke mechanical hypersensitivity. **(A)** The schematic describes pre-treatment of Schwann cells with vehicle followed by addition of GSK1016790A (GSK) or pre-treatment with either of two doses of the TRPV4 antagonist HC-067047 (HC), followed by activation of TRPV4 by addition of GSK. CM from the Schwann cell cultures was collected and filtered to remove drugs, and then resultant CM was injected into the mouse hind paw (created with BioRender.com). **(B)** CM from Schwann cell culture treated with TRPV4 agonist GSK evoked mechanical allodynia in mice, and the effect was attenuated when Schwann cells were pre-treated with TRPV4 antagonist HC; treatment *F*_(3, 16)_ = 12.7, *p* = 0.0002; ***p = 0.0001 at 1 h, ****p < 0.0001 at 3 h when mice injected with CM from vehicle + vehicle-treated Schwann cells are compared with mice injected with CM from vehicle + GSK (0.3 µM)-treated Schwann cells; *n* = 5 mice/group, two-way ANOVA, Dunnett's multiple-comparisons test. Data are expressed as percent change from vehicle. Note that percent change in withdrawal thresholds of mice injected with CM from HC + GSK-treated Schwann cells are not significantly different from mice injected with CM from vehicle + vehicle-treated Schwann cells. Data are presented as mean ± SEM.

Cancer-activated Schwann cells induce nociceptive behaviors in mice, which have been attributed to the release of nociceptive mediators from the activated Schwann cells (22, 34). To investigate whether TRPV4 stimulates the release of nociceptive mediators from Schwann cells, we prepared CM from cultured primary mouse Schwann cells. We pre-treated the cultures with vehicle or TRPV4 antagonist for 15 min, followed by incubation with a TRPV4 agonist for 105 min (Figure 6A). The Schwann cell CM was cleared of drugs by filtration and injected into the hind paw of wild-type mice. Behavioral responses were measured to mechanical stimulation with von Frey fibers. Mice injected with the CM from Schwann cell cultures treated with vehicle + 0.3 µM GSK1016790A displayed hypersensitivity to mechanical stimulation at 1 and 3 h, measured as the percent change from the vehicle-treated CM group (vehicle + vehicle) (Figure 6B). Treatment with CM from cultures pre-treated for 15 min with 3 or 10 µM HC-067047 reversed the mechanical allodynia. These findings suggest that TRPV4 activates Schwann cells to release mediators evoking mechanical hypersensitivity.

## 4 Discussion

We demonstrate that activation of TRPV4 (on Schwann cells) mediates mechanical sensitivity and oral cancer pain. TRPV4 is functionally expressed in Schwann cells, consistent with a previous report (19), and activated by mechanical stimulation. Activation of TRPV4 on Schwann cells elicits the release of pain mediators in CM. Although we have not determined the specific nociceptive mediators released by Schwann cells following TRPV4 activation, previous studies suggest the involvement of IL-6, CX3CL1, and TNF-α from Schwann cells in oral cancer pain (22, 23, 35, 36). TRPV4 activation leads to the release of IL-6 and TNF-α from colonic epithelial cells during chronic inflammation (37) and increased expression of endothelin following exposure of keratinocytes to UVB (12). IL-6, TNF-α, and endothelin are established (oral cancer) pain mediators (22, 23, 38–40).

TRPV4 on neurons has been implicated in mediating pain associated with temporomandibular disorder, pulpitis, and visceral pain, inflammatory conditions in which expression of TRPV4 is upregulated on neurons (41–43). While we have previously reported increased expression of TRPV1 and TRPA1 in TGs of mice with tongue cancer (31, 44), we find that TRPV4 was neither abundantly expressed in nerves innervating murine tongue cancer nor in the TGs of mice with tongue cancer. A similar lack of increased TRPV4 expression in TG neurons was observed in a rat oral tongue cancer model, in which pharmacologic antagonism of TRPV4 reduced mechanical sensitivity (45), as observed here. Rather, the authors reported increased phosphorylation of TRPV4 on TG neurons (45). We also found expression of TRPV4 to be low in naïve mice. This observation is in agreement with published data from two independent studies based on single-cell RNA sequencing of mouse TGs, which reported that only a small proportion of neurons (0.25% and 6%) express *Trpv4* (46, 47). We cannot rule out a contribution of TRPV4 expressed in neurons to oral cancer pain; however, clinical experience finds that oral cancer pain is not alleviated by NSAIDs, anti-inflammatory drugs commonly used to treat temporomandibular joint pain (48), for example, suggesting the etiology of oral cancer pain differs from painful conditions associated with inflammation and upregulation of neuronal TRPV4.

Thermal hypersensitivity in our HSC-3 oral cancer model was not reversed by the antagonism of TRPV4. We demonstrated that Schwann cells release mediators of mechanical sensitivity following TRPV4 activation, but we did not test whether CM from TRPV4 agonist-treated Schwann cells induced thermal hypersensitivity. Whether mediators released from Schwann cells following TRPV4 activation would evoke thermal sensitivity is an open question. On the one hand, the HSC-3 xenograft tumor microenvironment is complex, being primarily composed of cancer cells, fibroblasts, endothelial cells, and immune cells. Schwann cells are a minority and therefore may make lesser contributions to the numerous mechanical and thermal pain mediators that have been reported for the HSC-3 model (31, 32, 49). On the other hand, as discussed above, TRPV4 activation in colonocytes and keratinocytes increased expression of IL6, TNF-α, and endothelin, all reported to be mediators of mechanical and thermal pain (12, 37), leaving open the possibility that TRPV4-activated Schwann cells could release both mechanical and thermal pain mediators.

This study has certain limitations that should be considered. While we studied behavior in two oral cancer models, one cancer model was generated in immunocompromised mice. The Schwann cells used in our functional studies were isolated from the spinal nerve of naïve mice or human subjects with unknown disease conditions. Schwann cells are plastic, changing their phenotype to promote wound and nerve repair (50). Cancers reprogram tumor and peri-tumoral Schwann cells to a repair-like state (21, 51), raising the question of how oral cancer-associated Schwann cells might differ from the cells studied here. Further research is necessary to address these limitations.

In conclusion, our study identifies activation of TRPV4 in Schwann cells as a potential mediator of mechanically induced pain suffered by oral cancer patients. Improved pain management may be promoted by knowledge of the role of mechanosensitive ion channels in the cancer microenvironment.

## Data Availability

The datasets presented in this study can be found in online repositories. The names of the repository/repositories and accession number(s) can be found in the article/Supplementary Material.
