# Supplementary material for: TRPV4 activation in Schwann cells mediates mechanically induced pain of oral cancer
Source: Front Pain Res (Lausanne). 2025 Mar 12;6:1532885. doi: 10.3389/fpain.2025.1532885 (PMC11937083; doi:10.3389/fpain.2025.1532885)
Supplement: Supplementary file 1 [file Table1.docx]

Supplementary Material

**Supplementary Methods:**

**Immunohistochemical (IHC) staining of mouse tongue for TRPV4:**

Mice were sacrificed by isoflurane overdose and tongues were harvested, bisected longitudinally and fixed in 10% neutral buffered formalin. Tissues were dehydrated through graded ethanols and xylene and then processed into paraffin on a Leica Peloris II tissue processor. Embedded tissues were sectioned at 5 µm and processed for immunostaining of TRPV4 as described for the IHC staining of Schwann cell pellet (Methods 2.9 of manuscript text).

**RNA *in situ* hybridization (FISH) and subsequent immunofluorescence staining:**

RNA *in situ* hybridization was performed using RNAscope^TM^ Multiplex Fluorescent Reagent Kit v2 (Advanced Cell Diagnostics, ACD; Cat. #: 323100) with slight modifications from the manufacturer’s recommendations, as previously described (1). Briefly, trigeminal ganglia were fixed in 4% paraformaldehyde for 15 minutes on ice, washed in cold PBS, and submerged in 30% sucrose in 1x PBS at 4 ºC overnight. Tissue was embedded in Tissue-Tek OCT (Sakura Finetek USA, Inc.; Cat. #: 4583) and frozen over liquid nitrogen. Sagittal tissue cryosections (14 µm) were prepared on slides and sections were air dried at -20 ºC for 1 hour. Slides were washed in 1x PBS and then dehydrated at room temperature in 50%, 70%, 100%, 100% ethanol/PBS for 5 minutes each and allowed to dry for 5 minutes. Dehydration was followed by RNAscope™ hydrogen peroxide pretreatment of sections for 10 minutes at room temperature with the reaction stopped by washing twice in Millipore filtration system ultrapure water. RNAscope™ Protease IV solution was used to digest the sections for 30 minutes at room temperature and then washed in PBS twice. Sections were then incubated at 40 °C using the RNAscope™ HybEZ oven and humidity control tray with the following solutions: 1) target probe (mouse *Trpv4*; RNAscope™ Probe - Mm-Trpv4, Cat. #: 406071, ACD) or control probe (RNAscope™ 3-plex Negative Control, Cat. #: 320871, ACD) for 2 hours; 2) Amp 1 for 30 minutes; 3) Amp 2 for 30 minutes; 4) Amp 3 for 15 minutes; 5) HRP-C1 for 15 minutes; 6) Opal dye 650 (Akoya Biosciences; Cat. #: FP1496001KT) diluted 1:2000 in TSA buffer for 30 minutes; 7) HRP blocker for 15 minutes. After each of these incubation steps slides were washed twice for 2 minutes using RNAscope™ wash buffer prepared and diluted according to the manufacturer’s instructions at room temperature using a shaker.

After RNAscope, immunohistochemistry proceeded with permeabilization and blocking in 0.1% Triton-X, 5% normal goat serum mixed in PBS at room temperature for 1 hour. Following that, slides were incubated with chicken 𝛽-tubulin III antibody diluted (1:2000) in primary antibody solution (Abcam; Cat. #: ab107216, RRID: AB_10899689) overnight at 4°C. The following day slides were washed three times for 5 minutes each in PBS before being incubated in secondary antibody solution containing goat anti-chicken 488 (Thermo Fisher Scientific; Cat. #: A-11039, RRID: AB_2534096) diluted 1:1000. Slides were then washed five times for 10 minutes each in PBS before being mounted with DAPI Fluoromount-G (SouthernBiotech; Cat.#: 0100-20). Sections were imaged on a Leica SP8 confocal microscope with a 40x lens (NA= 1.3). Sequential scanning was performed with laser excitation (EX) and emission (EM) wavelengths (nm): DAPI: EX 405/EM 410-643, 𝛽-tubulin III: EX 488/EM 500-560, Probe: EX 638/EM 656-789. Images (16 bits, 1024x1024 pixels) were acquired with Z-step size of 0.33 µm. Analysis was performed in QuPath (2). Neurons were positively identified based on 𝛽-tubulin III staining and were counted as positive for *Trpv4* if they had more puncta than the maximum observed in control probe treated slides (>1 spot).

**References:**

1. Griffith TN, Docter TA, Lumpkin EA. Tetrodotoxin-Sensitive Sodium Channels Mediate Action Potential Firing and Excitability in Menthol-Sensitive Vglut3-Lineage Sensory Neurons. J Neurosci. 2019;39(36):7086-101.

2. Bankhead P, Loughrey MB, Fernandez JA, Dombrowski Y, McArt DG, Dunne PD, et al. QuPath: Open source software for digital pathology image analysis. Sci Rep. 2017;7(1):16878.

**Supplementary Figures**

**Supplementary Figure 1:** Immunohistochemical staining of a mouse tongue with TRPV4 antibody revealed TRPV4 immunoreactivity on the membrane of basal cells in the tongue epithelium. (**A**), von Ebner glands (**B**), and inner lining of blood vessels (**C**). No TRPV4 immunoreactivity was observed in nerve bundles (**D**). Black arrows indicate TRPV4 localization signal and red arrows indicate lack of TRPV4 signal in nerves.

**
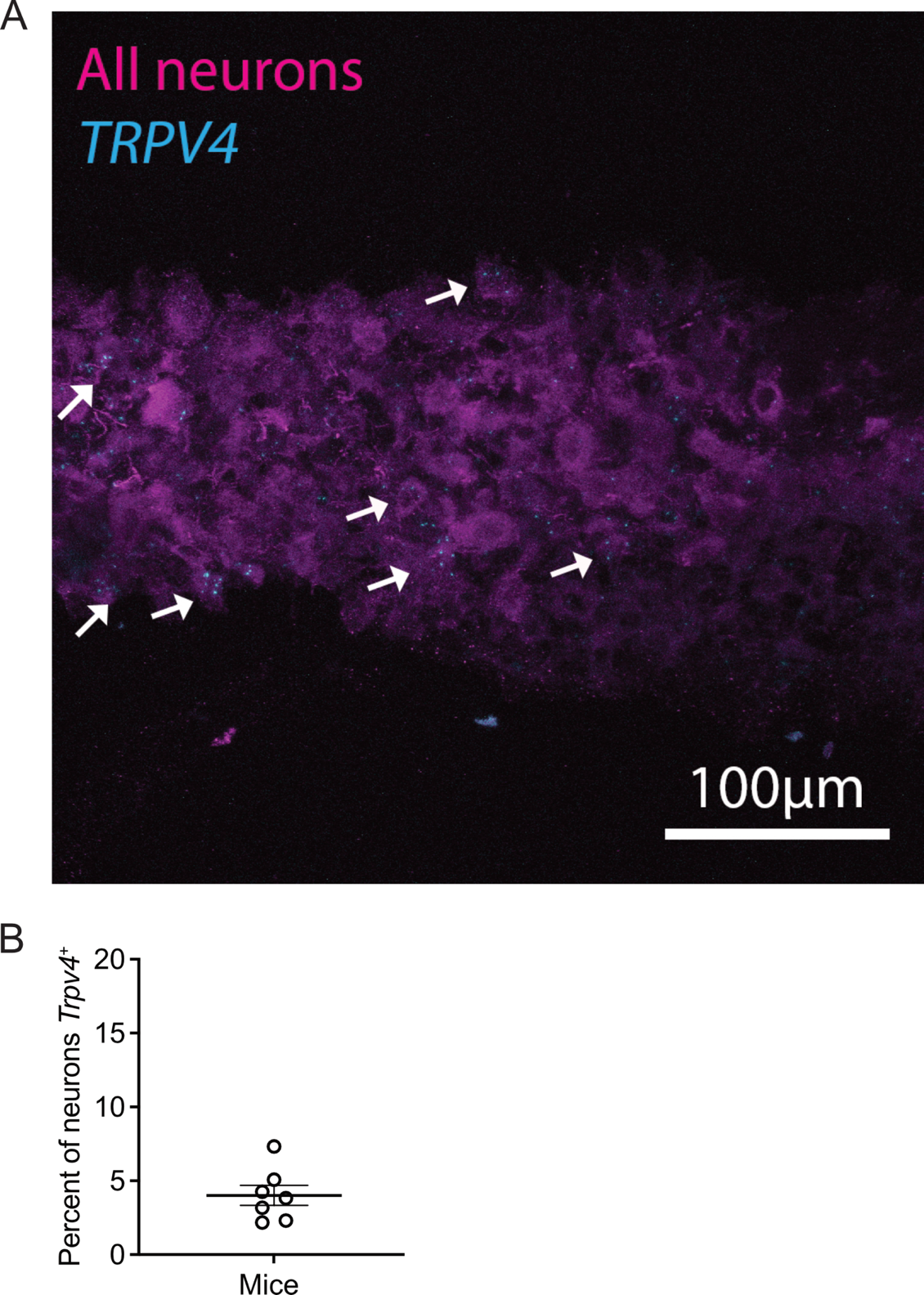
**

**Supplementary Figure 2**: Expression of *Trpv4* in the mouse trigeminal ganglia (TG). (**A**) Representative image of RNAscope *in situ* hybridization and fluorescence detection of *Trpv4* transcripts in a mouse TG. Shown are neurons identified by 𝛽-tubulin III (TUBB3) immunohistochemical reactivity (magenta) and *Trpv4* hybridization signals in cyan (white arrows). (**B**) Scatter plot showing percent of TG neurons that were *Trpv4^+^* (n=7 mice, 3 males and 4 females, no significant difference between sexes detected by unpaired two-tail t-test). The number of *Trpv4^+^* cells/mouse ranged from 9 - 47 of 414 - 1229 neurons counted/mouse). Data are expressed as mean ± S.E.M.

**Supplementary Table S1: Key resources**

| **Resource** | **Vendor** | **Identifier** |
| --- | --- | --- |
| **Animals** |  |  |
| C57BL/6J | Jackson Laboratory, Bar Harbor, ME | Cat. #: 000664, RRID:IMSR_JAX:000664 |
| NU/J | Jackson Laboratory, Bar Harbor, ME | Cat. #: 002019, RRID:IMSR_JAX:002019 |
| **Primary cells/cell lines** |  |  |
| Human oral tongue cancer cell line (HSC-3) | Japanese Collection of Research Bioresources Cell Bank | Cat. #: JCRB0623,  RRID: CVCL_1288 |
| Murine oral cavity cancer cell line (MOC2) | Kerafast, Boston, MA | Cat. #: EWL002-FP,  RRID: CVCL_ZD33 |
| Primary mouse Schwann cells | ScienCell Research Laboratories, Carlsbad, CA | Cat. #: M1700-57 |
| Spontaneously immortalized mouse Schwann cells | Applied Biological Materials Inc., Richmond, BC, Canada | Cat. #: 1970C3 |
| Primary human Schwann cells | Neuromics Inc., Minneapolis, MN | Cat. #: HMP303 |
| **Culture media** |  |  |
| HyClone Iscove's Modified Dulbecco's Medium | Cytiva Life Sciences, Wilmington, DE | Cat. #: SH30228.02 |
| Ham’s nutrient mixture F12 | Cytiva Life Sciences, Wilmington, DE | Cat. #: SH30026.01 |
| Spontaneously immortalized mouse Schwann cell medium (PriGrow III medium) | Applied Biological Materials Inc., Richmond, BC, Canada | Cat. #: TM003 |
| Primary mouse Schwann cell medium | ScienCell Research Laboratories, Carlsbad, CA | Cat. #: 1701 |
| Primary human Schwann cell medium | ScienCell Research Laboratories, Carlsbad, CA | Cat. #: 1701 |
| **Drugs** |  |  |
| GSK2193874 | Tocris Bioscience, Minneapolis, MN | Cat. #: 5106 |
| GSK1016790A | Selleck Chemicals LLC, Houston, TX | Cat. #: S8107 |
| HC-067047 | Selleck Chemicals LLC, Houston, TX | Cat. #: S6637 |
| Ionomycin | ThermoFisher Scientific, Waltham, MA | Cat. #: I24222 |
| Fura-2-acetoxymethyl ester (Fura-2 AM) | ThermoFisher Scientific, Waltham, MA | Cat. #: F1201 |
| Poly-D-lysine | ThermoFisher Scientific, Waltham, MA | Cat. #: A3890401 |
| Rat tail type I collagen | Corning Inc., Corning, NY | Cat. #: 354249 |
| ***In vivo* experiments** |  |  |
| PDI Alcohol Prep Pad | PDI Healthcare, Woodcliff Lake, NJ | SKU #: B60307 |
| Lo-Dose^TM^ U-100 Insulin Syringe | Becton, Dickinson and Company, Franklin Lakes, NJ | Cat. #: BD 329461 |
| Von Frey filaments | Stoelting Co., Wood Dale, IL | Cat. #: 58011 |
| Paw thermal stimulator | IITC Life Sciences, Woodland Hills, CA | Cat. #: 390G |
| **qRT-PCR** |  |  |
| RNeasy mini kit | QIAGEN Inc., Redwood City, CA | Cat. #: 74104 |
| NanoDrop Spectrophotometer | ThermoFisher Scientific, Waltham, MA | Cat. #: ND-8000 |
| High-Capacity cDNA Reverse Transcription kit | ThermoFisher Scientific, Waltham, MA | Cat. #: 4368813 |
| PowerUp^TM^ SYBR^TM^ Green Master Mix | ThermoFisher Scientific, Waltham, MA | Cat. #: A25742 |
| AriaMx real-time PCR system | Agilent Technologies Inc., Santa Clara, CA |  |
| PrimeTime™ qPCR Primer Assays | Integrated DNA Technologies Inc., Coralville, IA |  |
| **Immunohistochemical staining** |  |  |
| Leica BondRX automated stainer | Leica Biosystems, Deer Park, IL |  |
| Leica Biosystems ER2 solution | Leica Biosystems, Deer Park, IL | Cat. #: AR9640 |
| Leica BOND Polymer Refine Detection System | Leica Biosystems, Deer Park, IL | Cat. #: DS9800 |
| TRPV4 primary antibody | Abcam, Waltham, MA | Cat. #: ab231772 |
| Hamamatsu NanoZoomer 2.0 HT | Hamamatsu Photonics, Bridgewater, NJ |  |
| NYU Grossman School of Medicine OMERO Plus | Glencoe Software Inc., Seattle, WA |  |
| NIH ImageJ |  | RRID: SCR_003070 |
| **Ca^2+^ imaging** |  |  |
| FlexStation 3 Microplate Reader | Molecular Devices, San Jose, CA |  |
| **Electrophysiology** |  |  |
| 5 mm round glass coverslips | Warner Instruments, Hamden, CT | Cat. #: 64-0700 |
| Patch electrodes | Sutter Instrument, Novato, CA | Cat. #: BF120-69-7.5 |
| Micro-pipette puller | Sutter Instrument, Novato, CA | Cat. #: P-2000 |
| Patch clamp recording chamber | Warner Instruments, Hamden, CT | Cat. #: 64-0381 |
| Axopatch 200B amplifier | Molecular Devices, San Jose, CA |  |
| Digidata 1440A digitizer | Molecular Devices, San Jose, CA |  |
| pClamp^TM^ 10 software | Molecular Devices, San Jose, CA |  |
| Piezo servo controller | Physik Instrumente, Auburn, MA | Cat. #: E-709.SRG |
| **Schwann cell conditioned media collection** |  |  |
| Protein concentrators PES | ThermoFisher Scientific, Waltham, MA | Cat. #: 88515 |
| **Statistical analysis** |  |  |
| GraphPad Prism 9 | GraphPad Software, Inc., San Diego, CA | RRID:SCR_002798 |

**Supplementary Table S2: Primers for qRT-PCR**

| **Gene** | **PrimerBank ID** | **Forward primer**  **(5’→3’)** | **Reverse primer**  **(5’→3’)** |
| --- | --- | --- | --- |
| *Trpv4* | 269784722c1 | AAACCTGCGTATGAAGTTCCAG | CCGTAGTCGAACAAGGAATCCA |
| *Trpa1* | 29244298a1 | GTCCAGGGCGTTGTCTATCG | CGTGATGCAGAGGACAGAGAT |
| *Trpv1* | 47825363c1 | CCGGCTTTTTGGGAAGGGT | GAGACAGGTAGGTCCATCCAC |
| *Sox10* | 226423936c2 | AGGTTGCTGAACGAAAGTGAC | CCGAGGTTGGTACTTGTAGTCC |
| *S100b* | 6677839a1 | TGGTTGCCCTCATTGATGTCT | CCCATCCCCATCTTCGTCC |
| *Plp1* | 88196798c1 | ATGGGCTTGTTAGAGTGTTGTG | GTACCAGTGAGAGCTTCATGTC |
| *Gfap* | 30692526a1 | CGGAGACGCATCACCTCTG | AGGGAGTGGAGGAGTCATTCG |
| *Mbp* | 199051a1 | AATCGGCTCACAAGGGATTCA | TCCTCCCAGCTTAAAGATTTTGG |
| *Gusb* | 6754098a1 | GGCTGGTGACCTACTGGATTT | GGCACTGGGAACCTGAAGT |
